# Markers of kidney tubular and interstitial injury and function among sugarcane workers with cross-harvest serum creatinine elevation

**DOI:** 10.1136/oemed-2021-107989

**Published:** 2021-12-31

**Authors:** Erik Hansson, David H Wegman, Catharina Wesseling, Jason Glaser, Zachary J Schlader, Julia Wijkström, Kristina Jakobsson

**Affiliations:** 1 La Isla Network, Washington, DC, USA; 2 School of Public Health and Community Medicine, Institute of Medicine, University of Gothenburg Sahlgrenska Academy, Gothenburg, Sweden; 3 University of Massachusetts Lowell, Lowell, Massachusetts, USA; 4 Unit of Occupational Medicine, Institute of Environmental Medicine, Karolinska Institutet, Stockholm, Sweden; 5 Department of Kinesiology, Indiana University Bloomington School of Public Health, Bloomington, Indiana, USA; 6 Division of Renal Medicine, Department of Clinical Science, Intervention and Technology, Karolinska Institutet, Stockholm, Sweden; 7 Occupational and Environmental Medicine, Sahlgrenska University Hospital, Gothenburg, Sweden

**Keywords:** occupational health, physical exertion, kidney diseases, climate

## Abstract

**Objectives:**

Serum creatinine (SCr) is a routine marker of kidney injury but also increases with dehydration and muscular work. This study was to elucidate whether increase in SCr is associated with more specific markers of kidney tubular and interstitial injury and function, during prolonged heat stress among workers at high risk of chronic kidney disease of non-traditional origin (CKDnt).

**Methods:**

Urine monocyte chemoattractant protein-1 (MCP-1), kidney injury molecule-1 (KIM-1), calbindin, glutathione S-transferase-π (GST-π), clusterin, interleukin 18 and albumin, fractional excretion of potassium (FEK), blood haemoglobin, serum potassium, ferritin and erythropoietin were measured before and after harvest in a sample of 30 workers with a ≥0.3 mg/dL SCr increase across harvest (cases), and 53 workers with stable SCr (controls).

**Results:**

Urine MCP-1 (p for differential cross-harvest trend <0.001), KIM-1 (p=0.002), calbindin (p=0.02), GST-π (p=0.04), albumin (p=0.001) and FEK (p<0.001) increased in cases, whereas blood haemoglobin (p<0.001) and serum erythropoietin (p<0.001) decreased.

**Conclusion:**

Several markers of tubular and interstitial injury and function changed as SCr increased across a harvest season, supporting the use of SCr as an indicator of kidney injury in physically active workers regularly exposed to heat stress. Repeated injury similar to that described here, and continued work under strenuous and hot conditions with similarly elevated injury markers is likely to worsen and possibly initiate CKDnt.

**Key messages**

**What is already known about this subject?**

- Sugarcane workers in Mesoamerica are at high risk of chronic kidney disease and often experience increasing serum creatinine levels during harvest, something which may be a sign of acute kidney injury, or simply a sign of physiological processes related to physical work and dehydration.

**What are the new findings?**

- Increased serum creatinine levels were associated with increasing urine levels of several markers of reduced tubular function and injury, and reduced erythropoietin and haemoglobin levels.

**How might this impact on policy or clinical practice in the foreseeable future?**

- The results further support that kidney injury occurs among seemingly well sugarcane workers, strengthening the need for research and action aiming to understand, rapidly detect and prevent kidney injury among heat-stressed workers.

## Introduction

Workers in Mesoamerica regularly exposed to heat stress, such as sugarcane cutters, suffer from high rates of chronic kidney disease of non-traditional origin (CKDnt).^1^ There is a need for early detection of cases of kidney injury in populations at high risk of CKDnt to reduce risk, if possible. In our intervention studies at a sugarcane plantation in Nicaragua, we have used a 0.3 mg/dL elevation of early morning serum creatinine (SCr) across the 6-month interval between pre-harvest baseline and end-harvest samples as the main outcome.^2 3^ For epidemiological study purposes, we named this outcome incident kidney injury (IKI), to differentiate it from the clinical diagnosis acute kidney injury (AKI), which is defined by shorter time interval (48 hours)^4^ than is feasible to routinely monitor in occupational or community settings.

However, SCr has potentially important limitations as a marker of kidney injury among physically active and heat stress exposed workers.^5^ Specifically, increases in muscle mass and/or muscle breakdown may increase SCr levels independent of kidney injury. Further, creatinine excretion via glomerular filtration may temporarily be reduced by exercise and dehydration, reflecting a pre-renal physiological response rather than kidney injury. The relevance of monitoring changes in SCr over a few months should thus be critically assessed, as that is the outcome used in most longitudinal studies of populations at risk of CKDnt.^6 7^

There is emerging evidence that increases in SCr levels over a few months have implications for reductions in glomerular filtration rate (GFR) over a longer time perspective in populations at risk of CKDnt.^6–8^ It is important to understand whether this increase in SCr also indicates injury to the renal tubules and interstitium, which likely suffer the initial damage in CKDnt.^9^

Tubular cells reabsorb and excrete potassium in response to hormonal stimuli, thereby regulating body potassium homeostasis.^10^ Tubular injury may lead to hypokalaemia as potassium is lost with the urine.^10^ Hypokalaemia is a common finding in sugarcane cutters with IKI^11^ and CKDnt patients,^12–14^ with renal wasting of potassium reported among some.^12 14^ While albuminuria usually is indicative of glomerular filtration barrier dysfunction or damage, it has been suggested that low-grade albuminuria may arise when there is reduced tubular reabsorption of albumin filtered through healthy glomeruli.^15^

The hormone erythropoietin (EPO) is synthesised by interstitial fibroblasts in the kidney and promotes erythropoiesis.^16 17^ In response to tubular stress, injury and inflammation, EPO-producing cells may transform into myofibroblasts promoting progression of interstitial fibrosis and expressing less EPO, possibly leading to anaemia,^16 17^ which is a common finding in Mesoamerican sugarcane workers with recent SCr increase.^8 11 18^

Urinary biomarkers specifically indicating tubular injury have been studied in Mesoamerican populations at risk of CKDnt although patterns of change have been inconsistent. Urinary levels of kidney injury molecule 1 (KIM-1), neutrophil gelatinase-associated lipocalin, N-acetyl-β-d-glucosaminidase and interleukin 18 (IL-18) changed in different directions in different job groups over different periods of the harvest,^19 20^ and what these markers have to add to CKDnt research is at present unclear.^21^ For instance, which urine biomarkers best identify tubular kidney injury or progressive kidney disease is not well established, and the optimal marker likely varies by injured segment, time since onset of injury and underlying cause.^5 22^

This study aims to explore whether IKI is associated with markers of tubular injury as well as with altered function of tubulointerstitial cells (urine wasting of potassium and anaemia with EPO deficiency). Establishing if increased SCr in this context is associated with tubular injury and reduced tubular and interstitial function will help elucidate whether IKI, as defined above, is indicative of the type of kidney injury that can be considered to be an early stage of a progressive kidney disease, or merely represents physiological changes in SCr. The answer to this question is important for understanding the relevance of studies reporting SCr increase across short time frames such as months.

## Methods

### Study setting

This study was set at Ingenio San Antonio, a large sugar mill in Chichigalpa, Nicaragua, between November 2017 and April 2019.

As almost all IKI cases during sugarcane harvest that we have observed in our study occur in cutters,^2 3^ we only included cutters in the present substudy. On average, sugarcane cutters work at ~55% of maximum heart rate for 6–8 hours/day 6 days a week for 5–6 months.^23^ Wet-bulb globe temperatures generally exceed 30°C for a large proportion of the workday.^2 3^

A pre-employment health screen includes SCr and other blood or urine measurements. Workers with SCr >1.3 mg/dL, hyperuricaemia (serum uric acid >7.0 mg/dL in men), combined proteinuria and haematuria or anaemia (haematocrit <35%) are not supposed to be hired. At mid-harvest, workers are again screened using SCr and workers with SCr >1.3 mg/dL are put on a 2-week sick leave. They may return to work if SCr normalises. In addition, health promoters present in the field are responsible for ongoing monitoring of workers during the harvest and may refer unwell workers to the mill hospital, where SCr testing is routinely performed,^18^ something which may also result in sick leave. Aliquots of samples from mid-harvest and hospital are not preserved after testing.

### Substudy participant selection

Participants in the present substudy were male burned cane or seed cutters, aged <45 years, non-current smokers and had a baseline SCr <1.3 mg/dL and C reactive protein (CRP) <10 mg/L, forming a subsample from harvest 1 and 2 of the Adelante Initiative intervention study.^2 3^ Women were not included as no burned cane cutters and only one-fourth of seed cutters were women, and as there was only one female case in those two harvests.^2 3^ Smoking status was assessed by asking workers whether they currently smoke, and if not, whether they previously smoked. Workers with elevated CRP were excluded in order to exclude a small number of workers who may have had inflammatory reactions at the start of harvest, something which could interfere with our analysis especially of anaemia.

**Figure 1:**
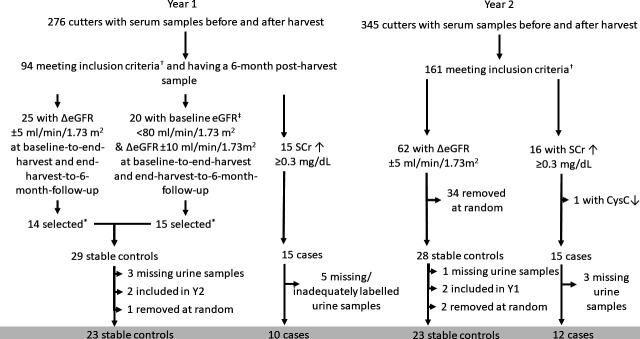
†Non-smoking men 18–45 years, baseline SCr <1.3 mg/dL and CRP <10 mg/L. ‡Cystatin C-based eGFR as this better reflects renal filtration and elimination of small proteins,^39^ something which is considered in a planned substudy using this material. *Those with normal (134–147 mmol/L) serum sodium levels in the first analysed batch were included to minimise potential influence of a previously reported mixing problem.^40^ Four selected cases were affected by this (sodium 131–133 mmol/L in the first aliquot analysis), these were included. CRP, C reactive protein; eGFR, estimated glomerular filtration rate; SCr, serum creatinine.

Cases were defined as having IKI if they had a SCr increase ≥0.3 mg/dL in morning serum samples from just before start (November) to end (April) of harvest.^2–4^ Controls were selected from cutters with stable estimated GFR (eGFR) across harvest (±5 in Harvests 1 and 2, and a group with±10 mL/min/1.73 m^2^ eGFR in Harvest 1) (figure 1). Control status was based on change in eGFR as this accounts better than changes in SCr for the non-linear association between SCr and GFR by taking into account baseline eGFR, age and sex, factors which all influence the association between SCr and GFR. We allowed for larger variation in cross-harvest eGFR change among controls with low baseline eGFR in Harvest 1 in order to obtain a larger overlap in absolute eGFR levels between cases and controls. Workers could participate in both harvests in the main study,^2^ but could be included in this substudy only in one of the harvests. No cases of IKI occurred in both years and no individual appeared as a case in 1 year and as a control in another year. Four workers were selected as controls in both harvests, but only included once. The harvest in which they were included was determined at random.

### Laboratory analyses

Urine and venous blood were sampled in the early morning hours both at baseline and end-harvest. After separating the blood cells from the serum, serum and urine samples were frozen in −80°C and transported to Sweden for later analysis.

#### Urine parameters

Urine potassium, albumin and creatinine were analysed at the Sahlgrenska University Hospital laboratory. We examined a subsample of markers considered indicative of tubular injury (monocyte chemoattractant protein-1 (MCP-1), KIM-1, calbindin, glutathione S-transferase-π (GST-π), clusterin and IL-18). The choice of this particular set of markers was determined by this set being available for analysis using a single multiplex assay (Bio-Plex Pro RBM Kidney Toxicity Panel I) consisting only of tubular injury biomarker assays. MCP-1 and KIM-1 are elevated in primarily proximal tubular injury^24 25^ and have been linked to biopsy-verified tubulointerstitial fibrosis and progression of kidney disease.^26 27^ Calbindin and GST-π are primarily expressed in the distal tubuli,^28 29^ but their association with long-term outcomes is less well understood. Clusterin and IL-18 are not considered segment-specific, but elevated urinary levels have been linked to worse eGFR trajectory.^27 30^

Urine concentration of MCP-1, KIM-1, clusterin, calbindin, GST-π and IL-18 were measured after urine samples had been diluted four times. IL-18 had 95% observations below limit of detection and therefore was excluded from further analysis. For the other five proteins, when concentrations were below the limit of detection, a value was assigned equal to the limit of detection divided by the square-root of two, a standard practice. All urine protein concentrations were then normalised to the urine creatinine concentration.

#### Serum parameters

SCr, CRP, ferritin and potassium were measured using a Cobas 701 instrument (Roche Diagnostics, Basel, Switzerland) by routine methods. Ferritin and EPO were measured only among IKI cases from year 1 and a random sample of controls from the same year, due to a limited amount of biobanked material. EPO was measured using R&D Systems ELISA kit. Samples for EPO analysis were diluted with one part as the available sample volume was low. Concentrations below the limit of quantification (LOQ) were assigned LOQ/√2, and then multiplied by the dilution factor, as were all other samples. Ferritin was measured in order to estimate iron reserves, as iron deficiency may be an alternative cause of anaemia. eGFR was calculated using the CKD Epidemiology Collaboration (CKD-EPI) equation for creatinine.^31^

#### Whole blood parameters

Baseline and end-harvest haemoglobin (year 2) or haematocrit (year 1) were measured on the day of sampling at the ISA laboratory using the whole-blood samples prior to freezing. Haematocrit was divided by 3 to estimate haemoglobin.

### Statistical analysis

Creatinine-corrected urine tubular injury protein, CRP and ferritin concentrations were transformed using Log_10_, to obtain distributions more closely resembling normal distributions. The fractional excretion of potassium (FEK) was calculated

(1) $$FEK=\frac{100*{\left[K\right]}_{urine}*{\left[creatinine\right]}_{serum}}{{\left[K\right]}_{serum}*{\left[creatinine\right]}_{urine}}$$

The statistical significance of differences between cases and controls at baseline, trends during harvest among controls and case–control differential cross-harvest trend was assessed using linear mixed models, including case status and baseline/end-harvest measurements as fixed effects with an interaction term, and a random intercept for each worker. Stata V.15 was used for all statistical analyses.

## Results

Twenty-two cases and 46 controls were analysed for tubular injury markers, after excluding 8 cases and 7 controls due to missing or possibly inadequately labelled urine samples for either baseline or end-harvest measurements. The controls thereby excluded were similar to controls included, while the cases thereby excluded had worse eGFR at end-harvest (median 41 vs 63 mL/min/1.73 m^2^) than those included and higher CRP at end-harvest (median 16.9 vs 6.4 mg/L). Cases and controls did not have different urine concentration or hydration status (urine creatinine and specific gravity) at baseline or different hydration status cross-harvest trajectories. Cases and controls had similar baseline age, eGFR, CRP and ferritin levels (table 1) as well as similar proportions from seed and burned cane cutter work groups, study year and workers sampled on Mondays (ie, after a rest day) at end-harvest.

**Table 1:** Descriptive data of workers with and without serum creatinine increase during sugarcane harvests at Ingenio San Antonio, Nicaragua, November 2017 to April 2019

		Cases (cross-harvest SCr increase by 0.3 mg/dL)				Controls (cross-harvest eGFR_crea_ change <10 mL/min/1.73m^2^)				Case–control comparison	
		n*	Baseline	End-harvest	Cross-harvest trend	n*	Baseline	End-harvest	Cross-harvest trend	Baseline	X-harvest differential trend
	Unit		Median (IQR)		p†		Median (IQR)		p†	p†	p†
Age	Years	22	26 (22–33)		NA	49	28 (23–37)		NA	0.25	NA
eGFR_creatinine_	mL/min/1.73 m^2^	22	95 (85–109)	63 (57–71)	<0.001	49	110 (81–123)	108 (81–123)	0.47	0.95	<0.001
U-Creatinine	mmol/L	22	6 (4–12)	6 (4–10)	0.63	49	8 (5–13)	5 (3–11)	0.02	0.59	0.35
USG	g/mL	22	1.012 (1.008–1.017)	1.012 (1.008–1.015)	0.66	49	1.012 (1.010–1.019)	1.010 (1.006–1.016)	0.26	0.72	0.80
S-Ferritin	µg/L	14‡	137 (83–238)	194 (114–316)	<0.001	28‡	101 (75–205)	99 (67–172)	0.10	0.40	<0.001
S-CRP§	mg/L	22	1.1 (0.6–3.3)	6.4 (1.0–15)	<0.001	49	0.7 (0.4–2.2)	1.0 (0.4–2.1)	0.25	0.18	<0.001
			**n (%)**				**n (%)**			**Case–control comparison, p¶**	
Cutter group	Burned cane	22	13 (59)			46	24 (52)			0.62	
	Seed cane		9 (41)				22 (48)				
Harvest	2017–2018	22	10 (45)			46	23 (50)			0.80	
	2017–2019		12 (55)				23 (50)				
End-harvest samples on a Monday		22	0			46	1 (2)			NA	

*See the Methods section for full description of selection of workers for each analysis.

†From linear mixed model, see the Methods section.

‡Ferritin levels were analysed in half the workers only and could not be determined for one case and one control due to insufficient sample volume.

§LOQ for CRP was 0.6 mg/dL.

¶Fischer’s exact test.

CRP, C reactive protein; eGFR, estimated glomerular filtration rate; LOQ, limit of quantification; S, serum; SCr, serum creatinine; U, urine; USG, urine specific gravity.

### Injury biomarkers

**Figure 2:**
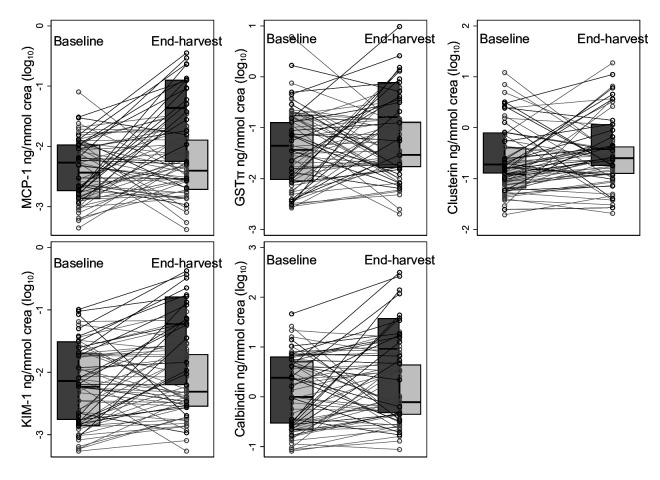
Tubular kidney injury markers before and after harvest. Cases (black) have cross-harvest SCr increase by >0.3 mg/dL and controls (grey) cross-harvest eGFR change <10 mL/min/1.73 m^2^ in either direction. eGFR, estimated glomerular filtration rate; GST-π, glutathione S-transferase-π; KIM-1, kidney injury molecule 1; MCP-1, monocyte chemoattractant protein 1; SCr, serum creatinine.

Urine MCP-1, KIM-1, GST-π and calbindin increased significantly more across harvest among cases (all p≤0.04). Urine clusterin doubled across harvest among both cases and controls (table 2, figure 2).

**Table 2:** Markers of kidney injury and function in workers with and without serum creatinine increase during sugarcane harvests at Ingenio San Antonio, Nicaragua, November 2017 to April 2019

					Cases (cross-harvest SCr increase by 0.3 mg/dL)				Controls (cross-harvest eGFR_crea_ change<10 mL/min/1.73 m^2^)				Case–control comparison	
	Unit	LOQ	% below LOQ	Unit/mmol creatinine	n*	Baseline	End-harvest	Cross-harvest trend	n*	Baseline	End-harvest	Cross-harvest trend	Baseline	X-harvest differential trend
						**Median (IQR)**		**p**†		**Median (IQR)**		**p**†	**p**†	**p**†
U-Clusterin	ng/mL	0.91	65	ng	22	0.19 (0.13–0.81)	0.38 (0.18–1.18)	0.23	46	0.12 (0.06–0.41)	0.25 (0.12–0.43)	0.10	0.23	0.96
U-Calbindin	ng/mL	1.6	45	ng	22	2.4 (0.3–6.4)	9.6 (0.5–38)	0.001	46	1.0 (0.2–5.1)	0.8 (0.4–4.4)	0.50	0.31	0.02
U-MCP-1	ng/mL	0.01	45	pg	22	5 (2–11)	43 (6–127)	<0.001	46	4 (1–11)	4 (2–11)	0.34	0.42	<0.001
U-KIM-1	ng/mL	0.01	35	pg	22	7 (2–31)	59 (6–163)	<0.001	46	6 (2–20)	5 (3–20)	0.23	0.23	0.002
U-GST-π	ng/mL	0.06	38	ng	22	0.05 (0.01–0.13)	0.17 (0.02–0.78)	0.01	46	0.04 (0.01–0.18)	0.03 (0.02–0.13)	0.98	0.91	0.04
S-K	mmol/L	NA	NA	NA	30	4.1 (3.8–4.4)	4.1 (3.5–4.5)	0.19	53	4.1 (3.8–4.4)	4.1 (3.9–4.3)	0.94	0.52	0.28
FEK	%	NA	NA	NA	22	10 (8–13)	20 (15–25)	<0.001	49‡	10 (9–13)	11 (9–16)	0.29	0.55	<0.001
U-Albumin	mg/L	3	66	g	22	0.43 (0.21–0.54)	1.42 (0.76–3.20)	<0.001	49‡	0.37 (0.27–0.61)	0.62 (0.41–1.14)	0.09	0.54	0.001
B-Hb	g/dL	NA	NA	NA	28§	13.6 (13.2–14.3)	12.2 (11.8–13.3)	<0.001	52§	14.4 (13.6–15.0)	14.1 (13.3–14.7)	0.23	0.05	<0.001
S-EPO	mIU/mL	0.4	13	NA	15	5.4 (2.3–6.1)	1.9 (0.7–3.3)	<0.001	21	4.9 (3.5–5.6)	6.0 (4.1–8.0)	0.04	0.52	<0.001

*See the Methods section for full description of selection of workers for each analysis.

†From linear mixed model, see the Methods section.

‡Three controls randomly excluded from injury marker analyses included in this analysis.

§Hb levels were missing for two cases and one control.

B, blood; eGFR, estimated glomerular filtration rate; EPO, erythropoietin; FEK, fractional excretion of potassium; GST-π, glutathione S-transferase-π; Hb, haemoglobin; K, potassium; KIM-1, kidney injury molecule 1; LOQ, limit of quantification; MCP-1, monocyte chemoattractant protein 1; S, serum; SCr, serum creatinine; U, urine.

### Function biomarkers

**Figure 3:**
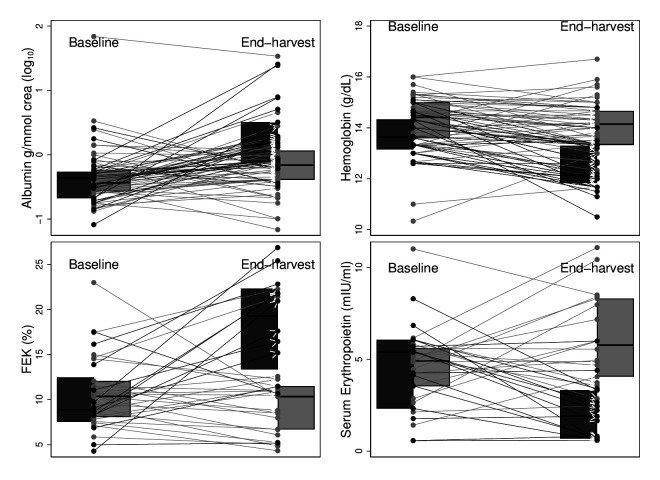
Kidney function markers before and after harvest. Cases (black) have cross-harvest SCr increase by >0.3 mg/dL and controls (grey) cross-harvest eGFR change <10 mL/min/1.73 m^2^ in either direction. eGFR, estimated glomerular filtration rate; FEK, fractional excretion of potassium; SCr, serum creatinine.

FEK doubled among cases and remained stable in controls (p for differential trend <0.001). Median haemoglobin decreased by approximately 1.5 g/dL in the case group, remaining largely stable among controls (p for differential trend <0.001) (table 2, figure 3). Cases had a decrease in EPO levels by approximately 65% (p for differential trend <0.001). Urine albumin increased among both cases and controls, but significantly more among cases (p=0.001).

## Discussion

### Interpretation

All markers of tubular injury or reduced tubular or interstitial function except clusterin and serum potassium worsened among IKI cases during harvest, indicating that cross-harvest increase in SCr is associated with tubular and interstitial kidney injury in this population.^9^

The non-concentration adjusted urine MCP-1 and KIM-1 levels among cases at end-harvest (online supplemental table 1) were comparable to those among 22 runners finishing the Boston marathon at an average speed of 10.5 km/hour.^32^ End-harvest levels of creatinine-adjusted KIM-1 among cases were not far below the median value for patients admitted to two general intensive care units (59 (IQR 6–163 pg/mmol Cr) vs 86 (IQR 37–210 pg/mmol Cr)),^33^ and median MCP-1 levels were approximately threefold higher than those measured in a cohort of diabetic patients with accelerated kidney disease progression (43 (IQR 6–127 pg/mmol Cr) vs 13 (IQR 9–21 pg/mmol Cr)).^34^ These findings support the magnitude of observed increases are likely clinically meaningful.

10.1136/oemed-2021-107989.supp1 Supplementary data

Interestingly, clusterin, the only tubular injury marker not increasing more among cases, has been found to be a sensitive marker of AKI induced by nephrotoxic medication such as calcineurin inhibitors in non-human primates.^35^ Pesticides proposed to have calcineurin inhibitory properties have been put forth as the cause of CKDnt,^36^ but considering that clusterin increase is not evident among cases, this seems unlikely to explain the IKI observed among the agricultural workers studied here.

The cross-harvest decrease in EPO concentrations and haemoglobin among those with IKI may reflect erythropoiesis limited by restricted EPO synthesis in kidney fibroblasts, as EPO increases in response to anaemia in individuals without CKD.^37^ Low ferritin levels (typically <30 µg/L) indicate iron deficiency, although concentrations increase with systemic inflammation. Cases did not have lower ferritin levels than controls before harvest, when neither they nor controls had signs of systemic inflammation (similar CRP levels (table 1)), indicating cases did not have lower iron reserves. During harvest, ferritin levels increased substantially in cases, consistent with ferritin being an acute phase reactant and with the inflammatory reaction seen in workers with IKI.^11^

Reduced EPO and haemoglobin levels can arise as interstitial EPO-synthesising cells transform to pro-inflammatory and -fibrotic myofibroblasts, a key step in the development of kidney interstitial fibrosis in CKD.^16 17^ Considering that anaemia has been associated with worse renal prognosis after AKI in this population^8^ and the role EPO-producing cells have in promoting interstitial fibrosis, reduced EPO synthesis points to kidney injury as an intermediary cause of anaemia and could also indicate a risk of progressive kidney disease. Measuring EPO levels is, however, costly, and the utility of monitoring more easily available haematological parameters (Hb or Hct) in conjunction with SCr for detecting kidney injury and early risk of CKDnt in populations at high risk of this disease should be further investigated.

Urine albumin levels were generally low, but increased more in cases during harvest, indicating renal injury. Determining whether this difference is due to reduced glomerular barrier function or reduced tubular albumin reabsorption among cases was not possible in this setting.

While FEK increased in cases across harvest, there was no overall decrease in serum potassium in this group. However, only 2% of total body potassium is in the extracellular compartment available for sampling, and the intracellular compartment may donate potassium to the extracellular in order to maintain stable serum levels. Mobilisation of intracellular stores of potassium may have buffered renal potassium losses and maintained stable serum potassium levels over the observed time period. Routine electrolyte supplementation received during work may also have influenced potassium homeostasis. As mentioned, hypokalaemia is a common finding in patients with established CKDnt,^12–14^ and the increased potassium losses seen here may be an early marker of tubular injury and risk of progressive kidney disease.

In year 2, we asked the workers present at end-harvest sampling if they had been on sick leave at any time during harvest. In this subsample 6/15 of the cases and 2/28 controls reported that they had been on sick leave during harvest—five cases and two controls due to SCr elevation and one case due to a cutting accident. One additional case and one control had SCr measurements above the company threshold for sick leave (1.3 mg/dL) at mid-harvest but reported no sick leave. Numbers are small, but taken together, this indicates that for some subjects rest after kidney injury was likely insufficient, or that new, undetected insults occurred later during harvest.

The workers in this study were sampled in the morning before a regular workday rather than in a care setting or after a rare event such as a marathon race. Considering that similar hot and strenuous conditions lead to a rise in tubular injury markers in humans in laboratory settings,^38^ and the strong association between physical workload and IKI,^2 3^ repetitive and prolonged heat stress exposure is likely to have implications for initiation of and failure to adequately resolve the various aspects of kidney injury documented in these workers. Although there remains a need for longitudinal research to determine whether these changes are only transient or are relevant in relation to risk of developing CKDnt, efforts to limit occupational heat stress in this population who are well known to be at high risk of CKDnt are warranted.^1^ Opportunities for early identification of kidney injury need to be available, as well as appropriate measures promoting recovery should kidney injury occur.

### Limitations

This study suffers from healthy worker selection bias. We can expect that those experiencing symptomatic heat illness who were tested during harvest for SCr increases to be at high risk of having been removed from the workforce and thus not included in end-harvest testing. This symptomatic group could be expected to have more severe kidney injury, possibly biasing the findings between IKI and markers towards null. That the workers with worse eGFR and higher CRP at end-harvest were less likely to provide urine samples could further increase this bias towards null.

Many of the tubular injury marker measurements were below level of quantification. Urine was diluted four times before analysis, an ad hoc procedure which turned out to be too extensive. A careful titration was not possible, due to a limited amount of urine available. Thus, IL-18 values, uniformly below the LOQ, could not be interpreted, and the measurement accuracies of the other markers were reduced, potentially biasing the findings between IKI and markers towards null.

The timing of injury in relation to measurement is another important consideration. While urinary levels of kidney injury markers tend to stabilise or even normalise the day after strenuous exercise,^32 38^ more complex temporal patterns are likely among sugarcane workers, with injury markers fluctuating with repetitive heat stress over a prolonged period of months. However, this remains unknown.

The association between SCr and tubular injury markers is likely to exist in other sugarcane mills and elsewhere where strenuous physical work is performed in high environmental heat. This study was conducted in healthy workers, selected not to have signs of kidney disease at start of harvest, at a sugarcane mill committed to improving working conditions through reducing heat stress^2 3^ and providing ad libitum access to purified, tested water and electrolyte solutions. Tubular injury marker trajectories are likely worse in settings where such efforts are not made. Working conditions were improved between years 1 and 2, but considering that the proportion of cases to controls included in this substudy was very similar between the years, this most likely has not introduced bias. It could have introduced bias if improved working conditions affected differently SCr and the other injury markers, and the proportion of cases and controls differed between years.

## Conclusion

In workers with IKI, defined as a >0.3 mg/dL pre-shift SCr increase over a harvest season, markers of tubular injury increased and signs of abnormal tubular and interstitial function appeared. This supports the continued use of repeated SCr measurements for assessing kidney injury among heat-stressed workers. Although the long-term relevance of IKI remains to be established in longitudinal epidemiological studies, the well-documented link between several of the injury markers and worse renal prognosis indicates that a mechanistic link between IKI and progressive kidney disease is likely.

## Data Availability

Data are available upon reasonable request.
