# Supplementary material for: Markers of kidney tubular and interstitial injury and function among sugarcane workers with cross-harvest serum creatinine elevation
Source: Occup Environ Med. 2021 Dec 31;79(6):396–402. doi: 10.1136/oemed-2021-107989 (PMC9120403; doi:10.1136/oemed-2021-107989)
Supplement: Supplementary data [file oemed-2021-107989supp001.pdf]

Table 1. Urine tubular injury markers among sugarcane cutters without adjustment for urine creatinine.

				Cases (cross-harvest SCr increase by 0.3 mg/dL)			Controls (cross-harvest $eGFR_{crea}$ change <10 ml/min/1.73m^2)		
				N	Baseline	End-harvest	N	Baseline	End-harvest
					Median (IQR)	Median (IQR)		Median (IQR)	Median (IQR)
Marker	Unit	LOQ	% <LOQ						
U-Clusterin	ng/ml	0.91	65%	22	LOQ (LOQ-5.5)	2.3 (LOQ-7.0)	46	LOQ (LOQ-3.2)	LOQ (LOQ-1.0)
U-Calbindin	ng/ml	1.6	45%	22	19 (LOQ-61)	78 (LOQ-197)	46	9 (LOQ-57)	LOQ (LOQ-39)
U-MCP-1	ng/ml	0.01	45%	22	0.03 (LOQ-0.13)	0.25 (0.03-1.35)	46	0.02 (LOQ-0.14)	LOQ (LOQ-0.13)
U-KIM-1	ng/ml	0.01	35%	22	0.07 (LOQ-0.35)	0.24 (0.02-1.62)	46	0.04 (LOQ-0.25)	0.02 (LOQ-0.20)
U-GST-π	ng/ml	0.06	38%	22	0.28 (LOQ-1.18)	0.90 (0.13-4.99)	46	0.39 (LOQ-1.49)	0.18 (LOQ-0.68)

MCP-1=Monocyte chemoattractant protein 1, KIM-1=Kidney injury molecule 1, GST-π =Glutathione S-transferase
